# Control of Phagocytosis by Microbial Pathogens

**DOI:** 10.3389/fimmu.2017.01368

**Published:** 2017-10-24

**Authors:** Eileen Uribe-Querol, Carlos Rosales

**Affiliations:** ^1^División de Estudios de Posgrado e Investigación, Facultad de Odontología, Universidad Nacional Autónoma de México, Mexico City, Mexico; ^2^Departamento de Inmunología, Instituto de Investigaciones Biomédicas, Universidad Nacional Autónoma de México, Mexico City, Mexico

**Keywords:** macrophage, neutrophil, bacteria, infection, inflammation, phagosome maturation, phagolysosome

## Abstract

Phagocytosis is a fundamental process of cells to capture and ingest foreign particles. Small unicellular organisms such as free-living amoeba use this process to acquire food. In pluricellular organisms, phagocytosis is a universal phenomenon that all cells are able to perform (including epithelial, endothelial, fibroblasts, etc.), but some specialized cells (such as neutrophils and macrophages) perform this very efficiently and were therefore named professional phagocytes by Rabinovitch. Cells use phagocytosis to capture and clear all particles larger than 0.5 µm, including pathogenic microorganisms and cellular debris. Phagocytosis involves a series of steps from recognition of the target particle, ingestion of it in a phagosome (phagocytic vacuole), maturation of this phagosome into a phagolysosome, to the final destruction of the ingested particle in the robust antimicrobial environment of the phagolysosome. For the most part, phagocytosis is an efficient process that eliminates invading pathogens and helps maintaining homeostasis. However, several pathogens have also evolved different strategies to prevent phagocytosis from proceeding in a normal way. These pathogens have a clear advantage to perpetuate the infection and continue their replication. Here, we present an overview of the phagocytic process with emphasis on the antimicrobial elements professional phagocytes use. We also summarize the current knowledge on the microbial strategies different pathogens use to prevent phagocytosis either at the level of ingestion, phagosome formation, and maturation, and even complete escape from phagosomes.

## Introduction

Phagocytosis, in pluricellular organisms, is a complex process for the ingestion and elimination of pathogens. It is also important for elimination of apoptotic cells, and for maintaining tissue homeostasis (1, 2). All cells may, to some extent, perform phagocytosis; however, in mammals, phagocytosis is the hallmark of specialized cells including monocytes, macrophages, dendritic cells, osteoclasts, eosinophils, and polymorphonuclear neutrophils—these cells are collectively referred to as professional phagocytes (3). Professional phagocytes eliminate microorganisms and present them to cells of the adaptive immune system. Phagocytosis can be divided into several main steps: (i) microbial recognition, (ii) phagosome formation, and (iii) phagolysosome maturation.

Phagocytosis initiates with the recognition and ingestion of microbial pathogens larger than 0.5 µm into a plasma membrane-derived vesicle, known as phagosome. This recognition is achieved through several receptors that recognize precise molecular patterns associated with microorganisms. These receptors then trigger signaling cascades that induce phagocytosis. Receptors on phagocytes can be divided into non-opsonic or opsonic receptors. Non-opsonic receptors can directly identify pathogen-associated molecular patterns (PAMPs) on the surface of the microorganisms. Opsonic receptors bind to host-produced molecules called opsonins. These molecules bind to microorganisms and mark them for ingestion. Opsonins include antibodies, complement, fibronectin, mannose-binding lectin, and milk fat globulin (lactadherin) (4).

**Figure 1:**
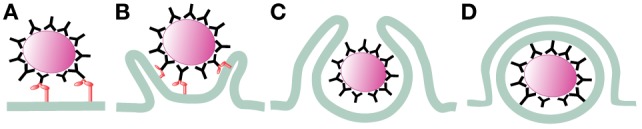
Initiation of phagocytosis. After receptor engagement **(A)** (2), the plasma membrane covers the microorganism to be ingested **(B)** (9, 10) and closes at the distal end **(C)** (11, 12), forming a vacuole where the microorganism is internalized **(D)**.

**Figure 2:**
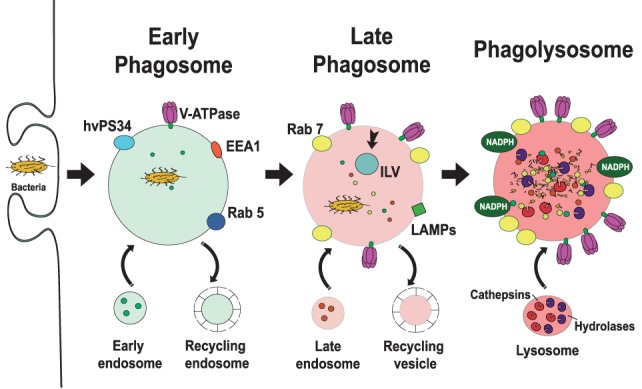
Phagosome maturation. The new phagosome quickly develops the characteristics of early endosomes, through a series of fusion and fission events with sorting and recycling endosomes (5, 6). *The early phagosome* is marked by the presence of the small GTPase Rab5 (13, 14), early endosome antigen 1 (EEA1) (15), and the class III PI-3K human vacuolar protein-sorting 34 (hvPS34) (16). The early phagosome also becomes a little acidic (pH 6.1–6.5) by the action of V-ATPase accumulating on its membrane (17, 18). *The late phagosome* is marked by the presence of Rab7 (19–21) and lysosomal-associated membrane proteins (LAMPs) (22, 23). Proteins that will be recycled are separated in sorting (recycling) vesicles, while proteins intended for degradation are eliminated in intraluminal vesicles (ILVs), directed into the lumen of the phagosome (24). The lumen of the late phagosome gets more acidic (pH 5.5–6.0), due to the action of more V-ATPase molecules on the membrane. *Phagolysosomes* are formed when late phagosomes fuse with lysosomes. Phagolysosomes are acidic (pH 5–5.5) and contain many degradative enzymes, including various cathepsins, proteases, lysozymes, and lipases. Other microbicidal component of the phagosome is the NADPH oxidase that generates reactive oxygen species (25).

After receptor engagement, the plasma membrane covers the microorganism to be ingested and closes at the distal end, forming a vacuole where the microorganism is internalized (Figure 1). This vacuole, the early phagosome, then fuses with endocytic vesicles and at the same time secretory vesicles are separated from it, transforming the early phagosome into a late phagosome. This dynamic process consists of sequential fusion and fission events between the new phagosome and endosomes, and it is known as “the kiss-and-run” model (5). Later, the intermediary phagosome turns into a microbicidal vacuole, the phagolysosome, by fusing with lysosomes and changing its membrane and interior characteristics through a process named phagolysosome maturation (6). The results of this process are remodeling of the membrane, progressive acidification of the phagosome, and creation of an oxidative and degradative milieu (7, 8) (Figure 2).

The phagocytic process is usually very efficient and concludes with the destruction of the microorganism ingested. Nevertheless, several pathogens possess various anti-phagocytic strategies, which allow them to survive and escape phagocytes. These strategies can be directed to any step of the phagocytic process. However, most microorganisms interfere with phagosome maturation since the phagolysosome is the most destructive organelle. It is the purpose of this review to highlight the multiple anti-microbial effectors of professional phagocytes and to describe how various microbial pathogens hinder phagocytosis to continue the course of their infection.

## Initiation of Phagocytosis

### Microbial Recognition

The first step in phagocytosis is the detection of a microorganism by phagocytes. Microbial pathogens are recognized directly by receptors that bind PAMPs or indirectly by receptors that bind opsonins. Receptors that directly bind PAMPs are known as pattern-recognition receptors and among these receptors, we find lectin-like recognition molecules, such as CD169 and CD33; C-type lectins, such as Dectin-2, Monocyte-INducible C-type LEctin (Mincle), or DNGR-1; scavenger receptors (26), such as scavenger receptor A, which detects lipopolysaccharide (LPS) on some Gram-negative bacteria (27), and on *Neisseria meningitidis* (27); and Dectin-1, which is a receptor for fungal beta-glucan (28, 29). Mannose receptors bind to mannan (30), and CD14 binds to LPS-binding protein (31). Toll-like receptors (TLRs), although recognize microorganisms, do not function as phagocytic receptors (32), however, they can cooperate with other non-opsonic receptors to stimulate phagocytosis (33).

Opsonins are soluble molecules that bind to microorganisms, marking them for phagocytosis. Antibody [immunoglobulin (Ig)] molecules and complement components are important opsonins that induce efficient phagocytosis (2). The most studied opsonic phagocytic receptors are the Fc receptors (FcRs), and the complement receptors (CRs), respectively (34). FcγRs bind to the constant (Fc) portion of IgG (35–38), while FcαRs bind IgA antibodies (39). CRs, such as CR3, bind to iC3b deposited on the microorganism after complement activation (40). Crosslinking of FcγR on the surface of cells activates efficient phagocytosis and other effector functions. These effector functions, such as activation of the oxidative burst, cell degranulation, antibody-dependent cell-mediated cytotoxicity, and activation of genes for production of cytokines and chemokines, are aimed toward the destruction of pathogens and the induction of an inflammatory response that is beneficial during infections (37, 41, 42). CRs, such as the integrin α_M_β2 (also known as CD11b/CD18, CR3, or Mac-1), bind the complement component iC3b deposited on pathogens to promote phagocytosis (34, 43).

### Phagosome Formation

After phagocyte receptors engage a microorganism, signaling events are triggered to initiate phagocytosis. Important changes in membrane remodeling and the actin cytoskeleton take place leading to the formation of pseudopods that cover the microorganism. Lipids associate and dissociate from the membrane of phagosomes in an orderly fashion (44), and the small GTPases Rho, Rac, and cell division cycle 42 (Cdc42), which are important regulators of the actin cytoskeleton, get activated and recruited to the forming phagosome (9, 10). At the point of contact, a depression of the membrane (the phagocytic cup) is formed. Then, F-actin polymerization allows formation of pseudopods that surround the target microorganism and within few minutes, the membrane protrusions fuse at the distal end (11, 12, 45) to seal the new phagosome (Figure 1).

## Phagosome Maturation

The new phagosome rapidly changes its membrane composition and its contents, to become a microbicidal vacuole, the phagolysosome. This process is known as phagosome maturation. The mechanism for transferring endocytosed material from endosomes to lysosomes is complex and not completely described. Four hypotheses have been proposed to explain the process of phagolysosome formation [reviewed in Ref. (46, 47)]. These include a maturation model where the endosome turns into a lysosome (48), a vesicular transport model where vesicles carry cargo from the endosome to the lysosome (49), a kiss-and-run model where endosomes and lysosomes engage in repeated transient fusions (50) and a direct fusion model where endosomes and lysosomes completely fuse giving rise to a hybrid compartment from which lysosomes reform (51, 52). Experimental evidence suggests that both the kiss-and-run and the complete fusion models contribute to the mechanism for delivery of endocytosed particles to the lysosome (53). The coordinated activities of endosomal sorting complex required for transport, homotypic fusion and vacuole protein sorting, and soluble *N*-ethylmaleimide-sensitive factor-attachment protein receptor protein complexes on the different vesicle membranes are required for efficient delivery of endocytosed macromolecules to lysosomes [reviewed in Ref. (54, 55)]. Phagosome maturation can be divided into three main stages, namely the early phagosome, the late phagosome, and the phagolysosome, as described later.

### Early Phagosome

The new phagosome quickly develops the characteristics of early endosomes, through a series of fusion and fission events with endosomes (5, 6). The early phagosome is marked by the presence of the small GTPase Rab5 (13, 14). This membrane GTPase regulates the fusion events between the phagosome and early endosomes by recruiting early endosome antigen 1 (EEA1) (15). Rab5 also recruits the class III PI-3K human vacuolar protein-sorting 34, which in turn, generates phosphatidylinositol 3-phosphate (16). This lipid then promotes recruitment of other proteins involved in phagosome maturation, including Rab7, a marker of late endosomes (19, 20). The early phagosome also becomes a little acidic (pH 6.1–6.5) by the action of V-ATPase accumulating on its membrane and also by transient fusions with more acidic vesicles (56). This V-ATPase translocates protons (H^+^) into the lumen of the phagosome using cytosolic ATP as an energy source (17, 18) (Figure 2).

### Late Phagosome

As maturation continues, Rab5 is lost, and Rab7 appears on the membrane. Rab7 mediates the fusion of the phagosome with late endosomes (21). At the same time, proteins that will be recycled are separated in sorting (recycling) vesicles, while proteins intended for degradation are eliminated in intraluminal vesicles, directed into the lumen of the phagosome (24). Furthermore, the lumen of the late phagosome gets more acidic (pH 5.5–6.0), due to the action of more V-ATPase molecules on the membrane (17) (Figure 2). Rab7 also recruits other proteins to the membrane. One such protein is Rab-interacting lysosomal protein (RILP), which brings the phagosome in contact with microtubules (57, 58), and lysosomes (57, 58). In addition, lysosomal-associated membrane proteins (LAMPs) and luminal proteases (cathepsins and hydrolases) are incorporated from fusion with late endosomes (7, 59) (Figure 2). LAMPs are a family of heavily glycosylated proteins that accumulate on the lysosomal membrane. They all contain a conserved intracytoplasmic tyrosine-based lysosome-targeting motif YXXφ (where φ represents a bulky hydrophobic residue) that directs the trafficking of the molecule through an endosome/lysosome pathway (60). LAMPs are fundamental in regulating membrane fusion events (61) and are required for fusion of lysosomes with phagosomes (22, 23).

### Phagolysosome

The late phagosomes gradually fuse with lysosomes, to become phagolysosomes, the definitive microbicidal organelles (Figure 2) (47, 53, 62). Phagolysosomes possess many sophisticated mechanisms directed to eliminate and degrade microorganisms. They are acidic (pH 5–5.5) thanks to the large number of V-ATPase molecules on their membrane (18) and contain many degradative enzymes, including various cathepsins, proteases, lysozymes, and lipases (17). Other microbicidal components of the phagosome are scavenger molecules, such as lactoferrin that sequesters the iron required by some bacteria (63), and the NADPH oxidase that generates superoxide $\left({\text{O}}_{2}^{-}\right)$ (25), and other reactive oxygen species (ROS) (64, 65) (Figure 2). Although the low pH is clearly microbicidal, it is important to note that phagosome acidification is highly regulated. The lysosomal pH may cycle between acidic and neutral conditions, allowing for the optimal activity of the different hydrolases (66). Within the hybrid degradative vesicle (phagolysosome) (46), most of these enzymes are active at pH 5–5.5; while in primary lysosomes that function as storage vesicles, the lower pH of 4.5 induces enzyme aggregation and inactivation (66).

## Antimicrobial Effectors

The phagolysosome is the definitive antimicrobial organelle. The arsenal at its disposal is large and complex. The major destructive strategies will be presented next.

### pH

**Figure 3:**
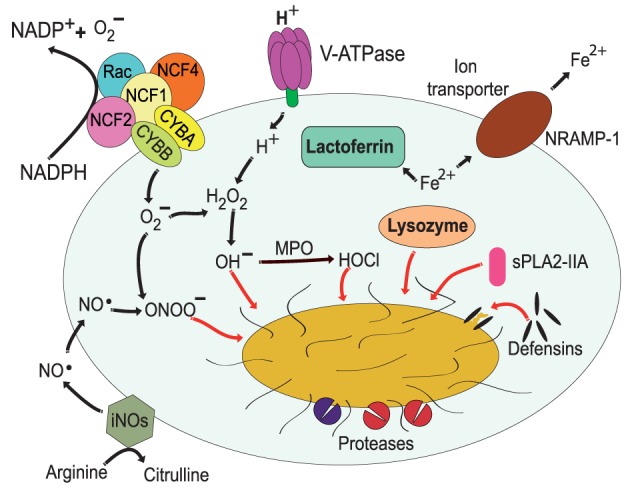
Antimicrobial effectors inside the phagolysosome. The most distinctive characteristic of phagolysosomes is their low pH. The V-ATPase translocates protons (H^+^) into the lumen of the phagosome (17, 18). The NADPH oxidase is an enzymatic complex formed by two transmembrane proteins, such as CYBB and CYBA, and three cytosolic components: NCF-4, NCF-1, and NCF-2 (68, 69). Rac is also required for efficient activation of the enzyme complex (70, 71). Myeloperoxidase (MPO) can transform H_2_O_2_ into hypochlorous acid (65). Nitric oxide radicals (NO^⋅^) are produced by the inducible nitric oxide synthase 2 (iNOS) (72), and NO^⋅^ reacts with ${\text{O}}_{2}^{-}$ to form peroxynitrite (ONOO^−^) (73, 74). Lactoferrin captures Fe^2+^ that is essential for bacterial growth (75), and the transporter natural resistance-associated macrophage protein 1 (NRAMP-1) takes Fe^2+^ out of the phagosome (76). Defensins are antimicrobial peptides that form multimeric ion-permeable channels on bacteria (77, 78). Cathepsins are lysosomal proteases (79, 80). Lysozyme (81, 82) degrades peptidoglycan, a primary building block of the cell wall of bacteria, and the type IIA secreted phospholipase A2 (sPLA2-IIA) (83) degrades anionic phospholipids such as phosphatidylglycerol, the main phospholipid component of bacterial membranes.

The most distinctive characteristic of phagolysosomes is their low pH. During the maturation process, the membrane of the phagosome accumulates more and more active molecules of V-ATPase. The molecular complex of this ATPase translocates protons (H^+^) into the lumen of the phagosome using cytosolic ATP as an energy source (17, 18) (Figure 3). The acidic content of the phagosome creates an adverse environment for most microorganisms. The low pH disrupts the normal metabolism of many bacteria and fungi and prevents the use of several essential microbial nutrients (67). In addition, the low pH is required for the activation of many hydrolytic enzymes, which will degrade the ingested pathogen. The V-ATPase also favors the generation superoxide $\left({\text{O}}_{2}^{-}\right)$ (25) by neutralizing the negative charges generated by the NADPH oxidase and by combining ${\text{O}}_{2}^{-}$ with H^+^ to generate other ROS (Figure 3) (as discussed later).

### Reactive Oxygen and Nitrogen Species

In addition to an acid environment, the phagolysosome can concentrate ROS to destroy microorganisms. Production of ROS is achieved by the NADPH oxidase (NOX2) on the membrane of phagosomes (68, 69, 84). The relevance of this antimicrobial mechanism is evident in patients with chronic granulomatous disease, who have mutations that result in malfunction of the NADPH oxidase. These patients suffer from severe recurrent infections that can cause death in many cases (85, 86). The NADPH oxidase is an enzymatic complex formed by two transmembrane proteins, CYBB (cytochrome *b*-245 heavy chain/gp91-phox) and CYBA (cytochrome *b*-245 light chain/p22-phox), that upon activation bring together three cytosolic components: NCF-4 (neutrophil cytosol factor 4/p40-phox), NCF-1 (neutrophil cytosol factor 1/p47-phox), and NCF-2 (neutrophil cytosol factor 2/p67-phox) (69, 87) (Figure 3). In addition, Rac1 and Rac2 are also required for efficient activation of the enzyme complex (70, 71). The oxidase transfers electrons from cytosolic NADPH to molecular oxygen to generate ${\text{O}}_{2}^{-}$ inside the phagosome (25). In the acid conditions of the phagosome, ${\text{O}}_{2}^{-}$ quickly dismutate into H_2_O_2_, which can then produce hydroxyl radicals (OH^−^) by a Fenton reaction (88) catalyzed by iron released from proteins in the phagosome (89). Also, myeloperoxidase can transform H_2_O_2_ into hypochlorous acid and chloramines (65) (Figure 3). In addition, ${\text{O}}_{2}^{-}$ can also react with nitric oxide (NO) to form peroxynitrite (ONOO^−^), both of which are highly reactive agents. The various forms of ROS are together efficient antimicrobial agents because they can damage proteins, lipids, and DNA of microorganisms in the phagosome (89).

In addition to ROS, phagocytes, such as macrophages, can also generate nitrogen-based radicals or reactive nitrogen species (RNS) that contribute to microbial destruction. Nitric oxide radicals (NO^⋅^) are produced by the inducible nitric oxide synthase 2 (iNOS or NOS2) (72). This enzyme is not present in the resting phagocyte and is only produced in response to proinflammatory agonists (90). NOS2 catalyzes the conversion of l-arginine and oxygen into l-citrulline and NO^⋅^ (Figure 3). Contrary to ${\text{O}}_{2}^{-}$, NO^⋅^ is produced on the cytoplasmic side of phagosomes, but it can diffuse across membranes and accumulate inside the phagosome (91). As mentioned earlier, once inside the phagosome, NO^⋅^ can react with ${\text{O}}_{2}^{-}$ to generate the highly toxic peroxynitrite (ONOO^−^) that can alter proteins and DNA of ingested microorganisms (73, 74). Also, NO^⋅^ can directly impair bacterial enzymes and affect microbial growth (92).

### Nutrient Capture

Not only the low pH and the oxidative conditions are used to harm the ingested pathogen but also a series of enzymes and peptides are delivered into the phagosome to limit its growth. Microbial growth can be limited by reducing the amount of essential nutrients inside the phagosome. Nutrients are eliminated from the phagosome by special capture molecules delivered into the phagosome or by transporters present on the phagosome membrane. Perhaps, lactoferrin is the best characterized capture molecule that prevents growth of some bacteria (75). Lactoferrin is a non-heme Fe^2+^-binding glycoprotein present in the specific granules of neutrophils (93), and it can be delivered into the phagolysosome. In there, lactofferin captures Fe^2+^ that is essential for bacterial growth (75, 94) (Figure 3). Other metals, such as Mn^2+^, are also important for microbial growth. Thus, during maturation, phagosomes become gradually depleted of Fe^2+^ and Mn^2+^ by the action the transporter natural resistance-associated macrophage protein 1 (NRAMP-1; also known as SlC11A1) (76) (Figure 3). NRAMP-1 is a membrane protein present on phagolysosomes that transports divalent cations, such as Fe^2+^, Zn^2+^, and Mn^2+^ out of the phagolysosome. This transporter requires H^+^ ions to function, thus NRAMP-1 is more efficient in the acid environment of the phagolysosome (76). Removal of these metal ions prevents microbial growth. However, some microorganisms present a mechanism to counteract the phagocyte function and retain these nutrients (see next section). Microorganisms secrete siderophores, specialized molecules that capture Fe^2+^ and target it for pathogen use (95). The phagocyte in turn presents yet another strategy to control microbial growth. The phagocyte protein lipocalin binds selectively catechol type siderophores expressed by some bacteria, such as *Escherichia coli* and *Mycobacterium tuberculosis*. Consequently, the Fe^2+^-loaded siderophore is still sequestered from the bacteria (96, 97).

### Microorganism Destruction

As described earlier, the phagolysosome interior is an inhospitable environment for most microorganisms. Enzymes and peptides delivered to the phagolysosome have potent antimicrobial effects by destroying the different components of the microbial cell.

Antimicrobial peptides are small (<10 kDa), cationic, and amphipathic polypeptides, often broadly classified based on structure (82, 98). In phagocytes, the main types include defensins (disulfide-stabilized peptides) (77, 78) and cathelicidins (α-helical or extended peptides) (99). Defensins are subdivided into α and β groups and are stored in primary granules of neutrophils. These peptides disrupt the integrity of pathogens by attaching to negatively charged molecules on their membrane. Defensins form multimeric ion-permeable channels that cause membrane permeabilization on both Gram-positive and Gram-negative bacteria (100) (Figure 3). Cathelicidins are stored in secondary granules of neutrophils as inactive precursors. They are then converted into active antimicrobial peptides in the lumen of the phagosome by elastase (99), a primary granule enzyme (93). Active cathelicidins permeabilize the cell wall and inner membrane of Gram-positive bacteria (100). In particular, the cathelicidin LL-37 (hCAP) has drawn particular attention because of its multiple functions. Not only LL-37 works as a broad-spectrum antibiotic but also it has potent chemotactic and immunomodulatory properties (101). Neutrophil-produced LL-37 can be internalized by macrophages (102) and can induce phagocytosis of IgG-opsonized Gram-negative and Gram-positive bacteria by these phagocytes (103). LL-37 also promotes leukotriene B4 and thromboxane A2 generation by human monocyte-derived macrophages (104), thus regulating the inflammation response during infections. Recently, it was also found that macrophages could also produce LL-37. Mice deficient in the Cramp (cathelicidin-related antimicrobial peptide) gene, the murine functional homolog of human LL-37, had increased susceptibility to *M. tuberculosis*; and macrophages from these mice were unable to control *M. tuberculosis* growth in an *in vitro* infection model (105).

Among the many degradative enzymes, the cathepsins are perhaps the most extensively characterized group of lysosomal proteases. These are cysteine proteases that play a direct role in microbial killing by inducing the disruption of bacterial membranes (Figure 3). For example, cathepsins L and K were found to be involved in phagocytosis and non-oxidative killing of *Staphylococcus aureus* (80), while cathepsin D controlled *Listeria monocytogenes* intracellular growth (79), probably by degrading the pore-forming toxin listeriolysin O of *L. monocytogenes* and thus preventing bacterial escape from the phagosome (see next section).

The phagolysosome also contains many lysosomal hydrolases, which help destroy ingested pathogens (106). An important enzyme of this group is lysozyme, an antibacterial protein that can be expressed and secreted by several cell types (81, 82). Lysozyme degrades peptidoglycan, a primary building block of the cell wall of bacteria (Figure 3). By breaking the bonds between *N*-acetylmuramic acid and *N*-acetyl-d-glucosamine, lysozyme disrupts the peptidoglycan integrity (107), and then other enzymes can complete the lysis of bacterial cells. One such enzyme is the type IIA secreted phospholipase A2 (sPLA2-IIA) (Figure 3), which exhibits potent antimicrobial activity against Gram-positive and Gram-negative bacteria (83). This remarkable property is due to the unique preference of sPLA2-IIA for anionic phospholipids such as phosphatidylglycerol, the main phospholipid component of bacterial membranes. The importance of this mechanism is highlighted by the fact that transgenic mice overexpressing human sPLA2-IIA are resistant to infection by *S. aureus, E. coli*, and *Bacillus anthracis*, the etiological agent of anthrax (83). Thus, antimicrobial peptides and degradative enzymes work together in the lumen of the phagolysosome to completely degrade most phagocytized microorganisms (Figure 3).

## Microbial Control of Phagocytosis

The discussion presented earlier clearly shows that phagocytosis is an efficient process (1, 2, 4, 108, 109) that culminates with the generation of the phagolysosome and its very harsh environment for most microorganisms (6). Therefore, it is not surprising that many successful pathogens have evolved multiple strategies to prevent and/or inhibit phagocytosis (110, 111). These strategies include prevention of phagocytosis, interference of phagosome maturation, resistance to phagolysosome contents, and even physical escape from the phagosome. Our knowledge comes mainly from studies of important extracellular and intracellular pathogens, such as *S. aureus* (112–114), *M. tuberculosis* (115–117), and *L. monocytogenes* (118, 119). However, many other microbial pathogens also have tactics for interfering with phagocytosis. The mechanisms for controlling phagocytosis employed by these model pathogens, as we understand them today, will be described next. In addition, information available on microbial control of phagocytosis by some other pathogens will also be presented.

### Prevention of Phagocytosis

**Figure 4:**
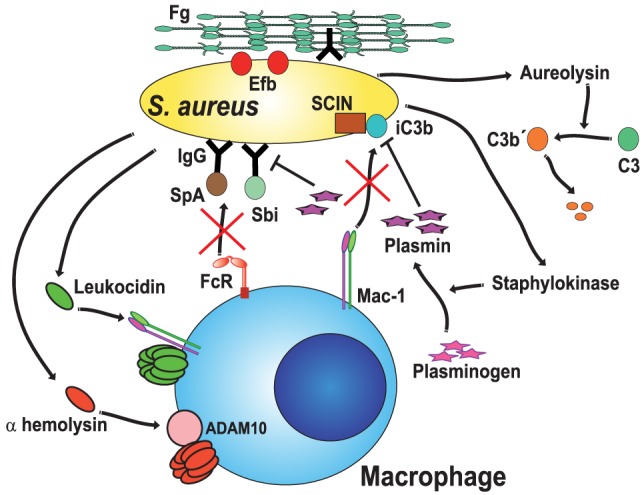
*Staphylococcus aureus* blocks opsonic phagocytosis. *S. aureus* secrete toxins, leukocidins (120, 125) and α-hemolysin (121), which induce membrane permeability by forming pores on the cell membrane. To be fully active, leukocidin A binds to the complement receptor Mac-1 (125), while α-hemolysin binds to protein ADAM10 (a disintegrin and metalloproteinase domain-containing protein 10) (127, 128). Staphylokinase converts host plasminogen to the active serine protease plasmin, which in turn degrades IgG or iC3b on the bacteria (127, 129). Protein A (SpA) (131) and staphylococcal binder of IgG (Sbi) protein specifically bind to the Fc region of IgG (132–134), blocking Fc receptor (FcR) engagement and activation. Aureolysin functions as a C3 convertase, leaving non-functional C3b′ fragments (135). Also, the staphylococcal complement inhibitor (SCIN) prevents complement activation on the bacteria (136). Finally, the extracellular fibrinogen binding protein (Efb) binds the serum protein fibrinogen (Fg), creating a proteinaceous shield that covers surface-bound opsonins (137, 138).

The best way to escape from the destructive power of phagocytosis would be just to prevent ingestion by phagocytes from happening. Some pathogens try just to do that by producing substances that extracellularly intoxicate phagocytes. *S. aureus* can secrete various membrane damaging toxins that will cause cell lysis and death. These toxins include the leukocidins (120) (named this way because they can kill leukocytes) and α-hemolysin (121) (Figure 4). Although there are different leukocidins, they all are dimer proteins (e.g., LukAB, LukED, HlgAB, HlgCB, and LukSF-PV) that induce membrane permeability by the formation of octameric β-barrel pores on the cell membrane (120, 122, 123). Interestingly, leukocidins do not attack any membrane indiscriminately. They must bind first to specific membrane receptors, and therefore, only cells with these receptors are targeted for intoxication (124, 125). For example, LukE binds to the chemokine receptor CCR5 on macrophages, marking these cells for lysis by the active leukocidin LukED (124, 126). Similarly, LukA binds only to the CD11b subunit of the CR Mac-1, which is expressed on both macrophages and neutrophils (125) (Figure 4). The α-hemolysin is another toxin from *S. aureus* that also forms pores on the membrane of macrophages. It uses phagocyte protein ADAM10 (a disintegrin and metalloproteinase domain-containing protein 10) as a receptor (127, 128), and then it assembles into a β-barrel pore of seven identical monomers across the cell membrane (129, 130) (Figure 4).

Phagocytosis is most efficient when microorganisms have been opsonized by antibodies or complement. Microorganisms have also evolved mechanisms to prevent opsonization. The first strategy displayed by *S. aureus* to block opsonization is simply to degrade opsonins. Some staphylococcal proteases seem capable of directly attacking opsonins. However, a more efficient instrument for this function is the protein staphylokinase, a bacterial plasminogen activator that converts host plasminogen to the active serine protease plasmin. Activated plasmin can then degrade IgG or C3b on the bacterial surface (139) (Figure 4). Another mechanism is to capture the opsonin, so that it does not bind to the bacteria. *S. aureus* Protein A is a well-known protein expressed on the bacterial cell wall. Protein A specifically binds to the Fc region of IgG, preventing the antibody from engaging FcγRs. In consequence, phagocytosis is effectively blocked (131) (Figure 4). In addition, Protein A can obstruct complement activation by the classical pathway, since the Fc portion of IgG is no longer accessible to the complement component C1q. This will result in less deposition of C3b on the bacteria (140). In addition to Protein A, most *S. aureus* strains express the Staphylococcal binder of IgG (Sbi) protein, which also binds to the Fc portion of IgG (132–134) (Figure 4). Inhibition of complement activation is an important strategy also used by *Staphylococcus*. The secreted metalloprotease aureolysin functions as an effective C3 convertase. Aureolysin cuts C3 in the α-chain at a different cleavage site from the C3 convertase, leaving C3a′ and C3b′ fragments (135). Unfortunately, the aureolysin-generated C3b′ fragment is rapidly degraded and not deposited on the bacteria (Figure 4). In addition, the staphylococcal complement inhibitor, a 10-kDa protein, can inhibit complement activation and efficiently prevent phagocytosis and killing of staphylococci (136) (Figure 4). As if all this were not enough, *S. aureus* can also hide the C3b deposited on its surface. The bacteria secrete the extracellular fibrinogen binding protein (Efb), which binds the serum protein fibrinogen (137). In this way, the bacterium creates a proteinaceous shield that covers the surface bound opsonin and prevents phagocytosis (137, 138) (Figure 4). This impressive array of anti-phagocytic effectors has been described for individual molecules. However, there is not enough information on when and how bacteria decide to use each one of them. The external elements that regulate the expression of each factor are not known. Novel techniques, such as expression profiling, should bring new light into these topics, as discussed later.

Another way to prevent ingestion by phagocytes from happening is to inactivate the cell machinery that creates the phagosome around the microorganism. Some pathogens have developed strategies to prevent actin polymerization and thus avoiding phagocytosis (141). The role of the actin cytoskeleton is fundamental for constructing a phagocytic cup and then extending membrane protrusions around the target particle. The small GTPase Rho family (10) controls formation of F-actin fibers required for phagocytosis. The GTPases Rho, Rac1, and Cdc42 act as molecular switches alternating between an active (GTP-bound) state and an inactive (GDP-bound) state (142, 143). For activation, they need to release GDP and replace it with GTP. This action is catalyzed by guanine nucleotide exchange factors (GEFs). Later, GTP is hydrolyzed to GDP returning the GTPase to its inactive state. This last step is enhanced through interactions with GTPase-activating proteins (GAPs). During phagocytosis, these GTPases are activated and recruited to the forming phagosome, where they activate nucleation-promoting factors such as Wiskott–Aldrich Syndrome protein (WASp) (144). WASp, in turn activates the actin-related protein 2/3 (Arp2/3) complex for actin polymerization (145, 146). As the new actin fibers grow, the plasma membrane is forced out, extending the membrane as pseudopodia around the particle to be ingested. Due to their central role in controlling actin dynamics, these small GTPases are the chosen target of some bacterial toxins. These toxins can alter the activity of the GTPases through covalent modifications or regulation of the nucleotide state. For example, the bacterium *Clostridium difficile*, which causes pseudomembranous colitis and is responsible for many cases of nosocomial antibiotic-associated diarrhea, produces two glycosylating exotoxins. Toxin A and toxin B modify Rho by glycosylation and inactivate its function. Rho inactivation causes disorganization of actin reducing phagocyte cell migration and phagocytosis (147). Similarly, the bacterium *Photorhabdus asymbiotica*, an emerging pathogen in humans, produces a toxin (PaTox) that tyrosine glycosylates Rho causing its inactivation. PaTox actions result in actin disassembly and inhibition of phagocytosis (148).

Another group of bacterial toxins regulate the nucleotide state and thus the function of the GTPases by functioning as GAPs or GEFs. For example, the enteropathogenic bacteria *Yersinia* spp. have type III secretion systems that inject Rho GAP toxins into cells. One such toxin (virulence factor) is YopO, which prevents Rac activation and in consequence prevents phagocytosis (149). Similarly, the Gram-negative bacteria *Pseudomonas aeruginosa*, an opportunistic pathogen that causes life-threatening infections in cystic fibrosis patients, burn victims, and immunosuppressed individuals, produces the type III virulence factor ExoS that is injected into cells. ExoS is a Rho GAP for Rho, Rac, and Cdc42 that causes the reorganization of the actin cytoskeleton by inhibition of Rac and Cdc42, and actin stress fiber formation by inhibition of Rho (150). An additional example recently described of pathogens disrupting Rho GTPase function comes from the opportunistic bacteria *Burkholderia cenocepacia* that has a propensity to infect cystic fibrosis patients. *B. cenocepacia* was shown to disrupt Rac and Cdc42 activation through perturbation of GEF function. Inactive Rac and Cdc42 led to inhibition of phagocyte function (151).

Besides bacteria, several fungal pathogens also display mechanisms for evading phagocytosis. *Candida albicans*, a commensal ascomycete, is part of the normal microbiota associated with mucosal tissues. It causes opportunistic infections, known as thrush, on superficial mucosas, and systemic infections, named candidiasis. *C. albicans* is normally phagocytized by macrophages, but it can decrease being recognized by phagocytes with a thick cell wall. The cell wall antigen, β-glucan is hidden among manno-proteins, thus reducing phagocytosis (152). In addition, *C. albicans* can limit phagocyte chemotaxis during transition from the yeast to the hyphal forms (153). Another fungus, *Aspergillus fumigatus*, also can mask antigenic proteins and carbohydrates to avoid recognition by phagocytes. RodA hydrophobin is a hydrophobic protein expressed on the surface of *A. fumigatus* conidia. This hydrophobin efficiently prevents recognition and phagocytosis (154). Similarly, the yeast basidiomycete *Cryptococcus neoformans* can also avoid recognition by macrophages. The basidiospores of *C. neoformans* produce a polysaccharide coat (capsule) that forms a thick barrier from phagocytes (155). This capsule can also be shed to prevent macrophage detection and phagocytosis (155). In addition, *C. neoformans* secretes antiphagocytic protein 1, a protein that binds to CR Mac-1 and inhibits phagocytosis (156).

### Interference with Phagosome Maturation

Once a microorganism is ingested, it will be exposed to the very harsh environment of the phagolysosome. Thus, many pathogens present strategies directed to avoid the formation of this final antimicrobial organelle. Phagosome maturation can be blocked at different points and there are examples of pathogens blocking acidification, reducing activation of the NADPH oxidase, and preventing phagosome to lysosome fusion. Perhaps the most studied example of inhibition of phagosome maturation occurs in *M. tuberculosis*. The first report was published more than 40 years ago (157), and since then several mycobacterial factors interfering with the process have been found, such as mannose-capped lipoarabinomannan (ManLAM), phosphatidyl-*myo*-inositol-mannosides (PIMs) (115, 158, 159), and trehalose-6,6′-dimycolate (TDM) (160).

**Figure 5:**
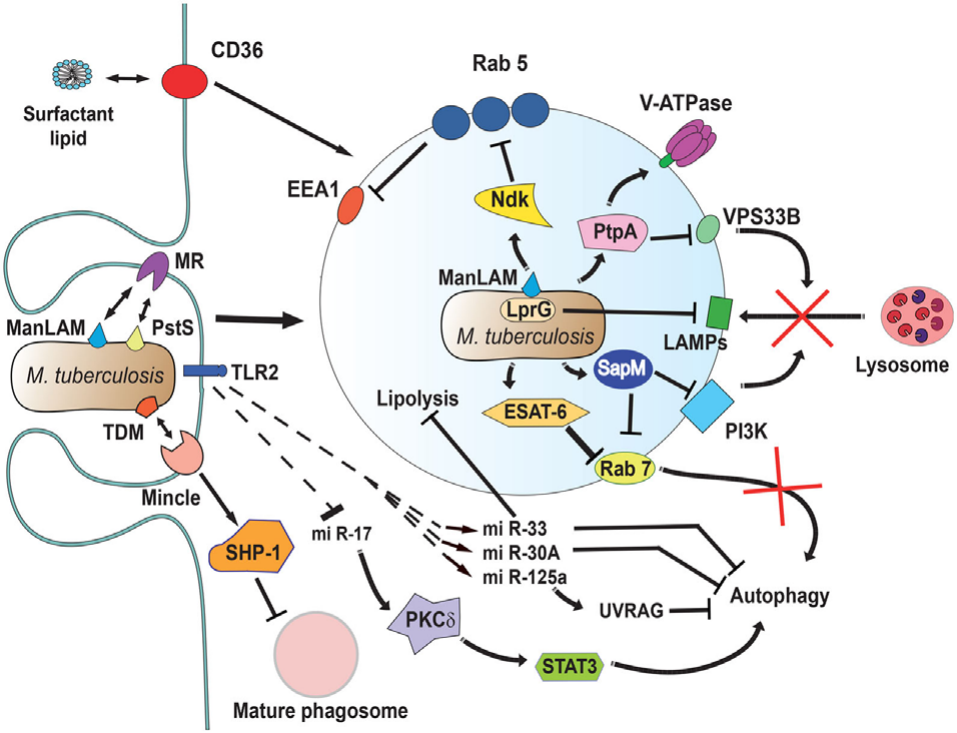
*Mycobacterium tuberculosis* interferes with phagosome maturation. *M. tuberculosis* inhibits acidification by preventing the accumulation of V-ATPase on the phagosome membrane (161), in part through the action of protein tyrosine phosphatase (PtpA) (162). PtpA also dephosphorylates human vacuolar protein sorting 33B (VPS33B) leading to the inhibition of phagosome-lysosome fusion (163). The nucleoside diphosphate kinase (Ndk) is a GAP for Rab5, and by inactivating this GTPase (164), it prevents recruitment of early endosome antigen 1 (EEA1) to the membrane (165). The lipoprotein LprG increases the surface-expression of mannose-capped lipoarabinomannan (ManLAM) (166) and can directly bind to lysosomal-associated membrane proteins (LAMPs) to modulate the traffic machinery of the cell (167, 168). Also, ManLAM (169) and the adhesin PstS-1 (170) bind the mannose receptor, which is involved in the lysosome fusion machinery by an unknown mechanism (171). The mycobacterial glycolipid TDM binds the receptor Monocyte-INducible C-type LEctin (Mincle) (172), activating the SH2-domain-containing inositol polyphosphate 5′ phosphatase (SHP-1) to interfere with phagosome maturation (160). The virulence factor early secretory antigenic target-6 (ESAT-6) inhibits recruitment of Rab7 to the phagosome membrane, preventing autophagy-mediated degradation (173). Also, the secretory acid phosphatase (SapM) direct binds to Rab7 (174) and prevents autophagosome-lysosome fusion (174). In addition, SapM can block the effects of phosphotidylinositol 3-kinase (PI3K) present on phagosomes (158). Upon infection, mycobacteria induce upregulation of several microRNAs (miRNAs) (175–177) and downregulation of others (178) to block autophagy. miR-125a targets UV radiation resistance-associated gene (UVRAG) (176) to block autophagy, while miR-17 activates a protein kinase Cδ (PKCδ)/signal transducer and activator of transcription 3 (STAT3) pathway to regulate autophagy (178). The miR-33 also inhibits fatty acid oxidation to support bacterial replication by a mechanism not yet described (177). How *M. tuberculosis* alters cell signaling to control miRNAs is not known, but the initial signal might come from TLR2 (176, 179). Finally, the scavenger receptor CD36 participates in surfactant lipid uptake by alveolar macrophages, and *M. tuberculosis* exploits this function for growth (180).

As mentioned earlier, one of the earlier features of phagosome maturation is the rapid and gradual acidification of the phagosome. The number of V-ATPase molecules increases on the phagosome membrane as the maturation process takes place. The low pH directly affects many pathogens (67), and it is also required for the activation of many hydrolytic enzymes. In the case of *M. tuberculosis*, acidification is inhibited by preventing the accumulation of V-ATPase on the phagosome membrane (161) (Figure 5). Although the complete mechanism is unknown, the *M. tuberculosis* secreted protein tyrosine phosphatase (PtpA) plays an important role. PtpA binds to subunit H of the macrophage vacuolar V-ATPase (162), and then it dephosphorylates human vacuolar protein sorting 33B (VPS33B) (163), leading to subsequent exclusion of the V-ATPase from the phagosome (Figure 5).

**Figure 6:**
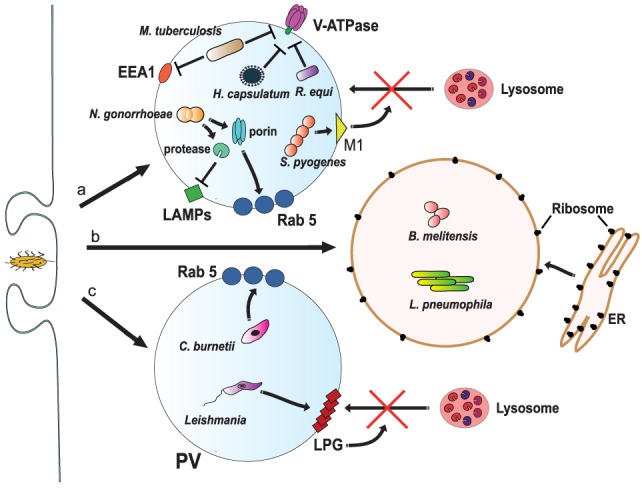
Inhibition of phagosome maturation. (a) Several pathogens, such as *Mycobacterium tuberculosis* (161), *Histoplasma capsulatum* (182), and *Rhodococcus equi* (183) inhibit acidification by preventing the accumulation of V-ATPase on the phagosome membrane. *M. tuberculosis* also blocks early endosome antigen 1 (EEA1) on the membrane (165), while *Neisseria gonorrhoeae* express a porin that induces large amounts of Rab5 (186) and also proteases that digest lysosomal-associated membrane proteins (LAMPs) (187). Another bacteria, *Streptococcus pyogenes*, express the virulence factor M1, which regulates vesicle trafficking (188). Each of these actions effectively will block lysosome fusion to the phagosome. (b) Other pathogens, such as *Legionella pneumophila* (189, 190) and *Brucella melitensis* (191), induce the rapid association of the phagosome with the endoplasmic reticulum (ER). (c) The bacteria *Coxiella burnetti* (192, 193), and the parasite *Leishmania* reside inside a phagolysosome-like vesicle known as parasitophorous vacuole (PV) that concentrates Rab5 on the membrane. *Leishmania* promastigotes also insert lipophosphoglycan (LPG) into the phagosome membrane (194). These actions, in consequence, prevent lysosome fusion (195).

The Gram-positive bacteria *Streptococcus pyogenes* blocks the V-ATPase activity through expression of surface proteins regulated by the virulence factor Mga (a transcription factor) (181). Similarly, *Rhodococcus equi*, Gram-positive bacteria that cause severe pneumonia in horses, and the dimorphic fungus *Histoplasma capsulatum* are also able to maintain a non-acidic phagosome by excluding the V-ATPase (182, 183) (Figure 6). Other pathogens can avoid acidification of phagosomes, including *Yersenia pestis*, the Gram-negative bacteria causing bubonic plague (184), and *C. albicans* (185), by mechanisms not completely described.

Phagosome maturation is also inhibited by interfering with the proper accumulation of molecules responsible for vesicle fusion, thus keeping the new phagosome with characteristics of an early phagosome. *M. tuberculosis* blocks phagosome maturation at a stage between the expression of Rab5 and Rab7, by preventing the delivery of the molecule EEA1 to the membrane (165) (Figure 5). This effect is mediated in part by the action of nucleoside diphosphate kinase (Ndk), which exhibits GAP activity toward Rab5 and Rab7. Ndk inactivates both Rab5 and Rab7 thus preventing recruitment of their respective effectors EEA1 and RILP and in consequence inhibits phagosome maturation and fusion with lysosomes (164) (Figure 5). This blockage also involves ManLAM (171), and it seems to require binding of ManLAM to the mannose receptor (169). Recently, the adhesin PstS-1, a 38-kDa mannosylated glycolipoprotein, was also found to bind the mannose receptor (170) (Figure 5). The connection between the mannose receptor and the lysosome fusion machinery is obscure. Because, capping of the ManLAM with mannose receptor was necessary during phagocytosis to maintain the blockade (169), it seems that the initial engagement of the mannose receptor directs, in an unclear manner, *M. tuberculosis* to a selective initial phagosomal niche, where other molecules can be excluded. Also, Mincle was recently identified as a receptor for the mycobacterial glycolipid TDM (172). Recruitment of Mincle by TDM coupled to IgG-opsonized beads during FcγR-mediated phagocytosis interfered with phagosome maturation (160). This inhibition involved the SH2-domain-containing inositol polyphosphate 5′ phosphatase (SHP-1) and the FcγRIIb (160), strongly suggesting an inhibitory downstream signaling of Mincle during phagosome formation (Figure 5). Without EEA1, delivery of the V-ATPase or enzymes such as cathepsin D does not take place (196). Therefore, the *M. tuberculosis*-containing phagosome is kept with a pathogen-friendly environment (Figure 5). Other microorganisms can also arrest phagosome maturation at early stages. For example, the Gram-negative bacteria *Neisseria gonorrhoeae* express a porin that induces phagosomes to keep larger amounts of Rab5 and low levels of Rab7 (186) (Figure 6). In addition, this bacterium also secretes proteases that digest LAMPs (187) (Figure 6). As mentioned earlier, LAMPs are fundamental for fusion of lysosomes to phagosomes (22), thus its degradation prevents formation of a mature phagolysosome (187). Similarly, the Gram-negative bacteria *Legionella pneumophila* intercepts vesicular traffic from endoplasmic reticulum (ER) (189) to create an organelle that allows the bacteria to have access to cysteine for survival (190) (Figure 6). This bacterium is the cause of Legionnaires’ disease, a severe form of pneumonia. When the bacteria are phagocytized, the phagosome is rapidly associated with mitochondria and the rough ER, thus getting decorated with ribosomes (197). This effect seems to be mediated by DotA, a bacterial product that is part of the type IV secretion system (T4SS) transporter. T4SS exports various bacterial effector proteins, including RalF, a GEF for the phagocyte ADP-ribosylation factor (ARF1) (198). Active ARF1 promotes vesicle traffic between the ER and the Golgi (199). Therefore, the ER-like phagosome does not get acidic and it does not fuse with lysosomes. Another example of phagosomes fusing with the ER is found in the Gram-negative bacteria *Brucella melitensis* (Figure 6). This bacterium is the etiological agent of brucellosis, a zoonotic infection that can cause muscle pain, fever, weight loss, and fatigue in people, but can also induce abortion and infertility in animals. In the macrophage cell line J774, *B. melitensis* alters vesicle trafficking (200) to create a modified phagosome known as a *Brucella*-containing vacuole (BCV) that fuses with the ER (191) (Figure 6). The mechanism for creating a BCV is not completely known, but it involves several virulence factors such as VirB, an element of the bacterial type III secretion system (191), and cyclic β-1,2-glucan, a cell wall component (201).

Since the phagolysosome is the most harmful organelle for microorganisms, many pathogens have mechanisms to prevent fusion of lysosomes with the phagosome. The best-known example is again *M. tuberculosis* that avoids lysosome fusion by maintaining an early phagosome (115) (Figure 5). The mechanism for this effect is multifactorial and complex. We only have a partial understanding of it with the identification of some key virulent factors involved. One such virulent factor is the lipoprotein LprG, which binds to lipoglycans, such as lipoarabinomannan (LAM), increasing the surface expression of LAM (166). A *M. tuberculosis* null mutant for LprG (Mtb ΔlprG) had lower levels of surface-exposed LAM and impaired phagosome–lysosome fusion (167). How LprG prevents phagosome–lysosome fusion is only partially known. It is possible that its effect is indirect *via* Ndk, which inactivates both Rab5 and Rab7 (164), or is direct by binding to LAMP-3 and modulating the traffic machinery in the host cell (168) (Figure 5). One more virulent factor is PtpA which, as mentioned earlier, dephosphorylates VPS33B, a regulator of membrane fusion events and leads to inhibition of phagosome–lysosome fusion (163) (Figure 5). Another way *M. tuberculosis* prevents phagosome–lysosome fusion involves inhibition of Rab7 recruitment to prevent autophagy-mediated degradation. The maturation of mycobacteria-containing autophagosomes into autolysosomes requires recruitment of Rab7, but this is blocked by the virulence factor early secretory antigenic target-6 (ESAT-6) (173) (Figure 5). Again, the molecular events for this blockage are not known. However, for another virulence factor of *M. tuberculosis*, the secretory acid phosphatase (SapM) the inhibition of autophagosome-lysosome fusion (202) is achieved *via* direct binding to Rab7 (174). Molecularly, Rab7 is blocked by SapM through its cytoplasmic domain preventing its involvement in autophagosome–lysosome fusion (174) (Figure 5). In addition, SapM is known to dephosphorylate phosphotidylinositol 3-phosphate present on phagosomes (158). This phospholipid is also required for membrane fusion events, thus SapM also prevents lysosome fusion in this manner (Figure 5).

*Mycobacterium tuberculosis* has also evolved other ways to prevent autophagy from happening. One recently described way is the activation or inhibition of cell host microRNAs (miRNAs). Upon infection, macrophages increased several miRNAs and inhibited pathways involved in autophagy. These miRNAs include miR-30A (175), miR-33 (177), and miR-125a (176) (Figure 5). At the same time, another miRNA, miR-17, is downregulated with the same result, blockage of autophagy (178). The signaling pathways affected by these miRNAs are only beginning to be described. For example, miR-125a targets UV radiation resistance-associated gene (UVRAG) (176) to block autophagy, while miR-17 activates a PKCδ/STAT3 pathway to regulate autophagy (178). Thus, inhibition of miR-17 leads also to reduce autophagy (Figure 5). How *M. tuberculosis* usurps cell host signaling pathways to alter expression of these miRNAs is not known. It seems, however, that the initial signal for this comes from TLRs (176) (Figure 5).

Similarly, *S. pyogenes* can also prevent lysosome fusion by expressing the virulence factor M1, which regulates vesicle trafficking (188) (Figure 6). M1 can also inhibit activation of the nuclear factor κB and in consequence reduce the macrophage inflammatory response (188). The Gram-negative bacteria *Coxiella burnetti*, the causative agent of Q fever, resides inside a large phagolysosome-like vesicle known as parasitophorous vacuole (192). This modified phagosome concentrates Rab5 on the membrane and avoids lysosome fusion (193) (Figure 6). The fungi *A. fumigatus* (203) and the parasitic protozoa *Leishmania* (204) seem also able to avoid being killed by macrophages by preventing fusion between phagosomes and lysosomes. In the case of *A. fumigatus*, the molecule dihydroxynaphthalene–melanin on the surface of the pathogen has been reported as responsible for altering vesicle fusion events (205). For *Leishmania*, the promastigote is efficiently internalized by receptor-mediated phagocytosis (204). Complement and mannose receptors participate in macrophage ingestion (195). Once internalized, promastigotes insert lipophosphoglycan (LPG) into the phagosome membrane. LPG inhibits depolymetization of F-actin (194), and in consequence prevents lysosome fusion (195) (Figure 6). This allows enough time for the promastigote to transform into the other life-cycle form, the amastigote, which can then replicate inside the phagosome.

### Resistance to Phagolysosome Contents

**Figure 7:**
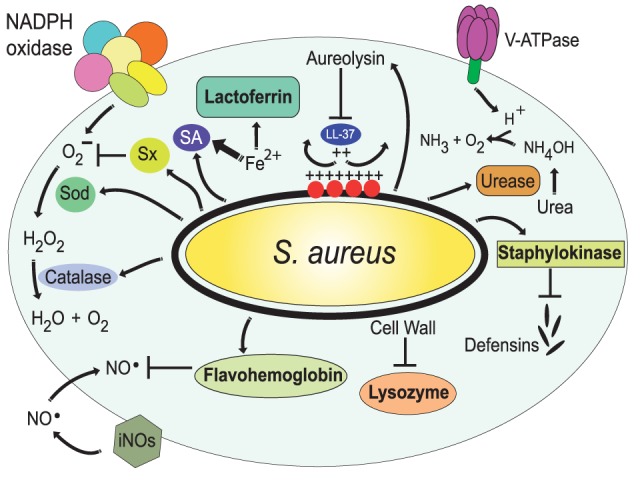
Resistance of *Staphylococcus aureus* to phagolysosome contents. The bacteria *S. aureus* modifies the composition of its cell wall to resist the action of lysozyme (206, 207) and alters the composition of its membrane, with l-lysine and lipoteichoic acids, to reduce the negative charge of the membrane (209, 210); thus resisting antimicrobial peptides, such as the cathelicidin LL-37. Also, it secretes staphylokinase and aureolysin to block α-defensins and LL-37, respectively (208, 211). In addition, *S. aureus* has the golden pigment staphyloxanthin (Sx), which works as an antioxidant (212), two super oxide dismutases (Sod) (213), and a catalase (214, 215) that together protect against reactive oxygen species. In addition, flavohemoglobin functions as an NO^⋅^ scavenger (216, 217). The bacterial urease catalyzes the hydrolysis of urea to form ammonia, resulting in pH neutralization (218). Finally, *S. aureus* produces siderophores (SA) (219, 220) that trap enough Fe^2+^ to allow bacterial survival.

In addition to preventing phagolysosome formation, pathogens also possess various mechanisms to resist the microbial components found in the phagolysosome lumen. A prominent example is *S. aureus* that can resist the lytic effect of lysozyme on the cell wall peptidoglycan. These bacteria express the enzyme *O*-acetyltransferase A (OatA), which causes O-acetylation of the peptidoglycan. This modification makes the peptidoglycan resistant to the muramidase activity of lysozyme (206, 207) (Figure 7). *S. aureus* also can block the action of antimicrobial peptides. First, the enzyme staphylokinase directly binds α-defensins, blocking almost completely their bactericidal effect (208) (Figure 7). Second, bacteria alter the composition of its membrane. Phosphatidylglycerol is modified with l-lysine, causing a reduction in the negative charge of the membrane (209). In addition, the cell wall is also modified by incorporation of teichoic acids and lipoteichoic acids (210), making it more positively charged. These modifications reduce interaction of α-defensins with the bacterial surface. Third, the metalloprotease aureolysin can degrade LL-37, an antimicrobial peptide with potent activity against staphylococci (211) (Figure 7).

Also, several pathogens express urease, an enzyme that catalyzes the hydrolysis of urea to form ammonia, resulting in the pH neutralization of the phagosome (Figure 7). Important examples of microorganisms using this strategy to survive in the phagosome are *S. aureus* (218), *Helicobacter pylori*, bacteria known for causing gastric and duodenal ulcers (221), *C. neoformans* (222), and *Coccidioides posadasii* (223).

The oxidative environment of the phagolysosome is also very damaging to most microorganisms. Yet, some pathogens have evolved ways to fight back the effects of ROS and RNS. For example, *S. aureus* has the golden pigment staphyloxanthin, which works as an antioxidant and prevents damage from peroxide (212) (Figure 7). Also, the protein SOK (surface factor promoting resistance to oxidative killing), that is expressed on the bacteria surface, was recently described as a virulence factor that blocks the effects of ROS (224). In addition, *S. aureus* express the enzymes super oxide dismutases, sodA and sodM, which convert ${\text{O}}_{2}^{-}$ into H_2_O_2_ (213), and the enzyme catalase (KatA), which breaks down H_2_O_2_ into oxygen and water (214, 215) (Figure 7). A phagocytized bacterium has also to prevent the effects of iNOS-derived RNS. *S. aureus* can detect NO^⋅^ by the two component system SsrAB (225), which regulates the expression of the gene hmp coding for a flavohemoglobin that functions as an NO^⋅^ scavenger (216, 217) (Figure 7).

**Figure 8:**
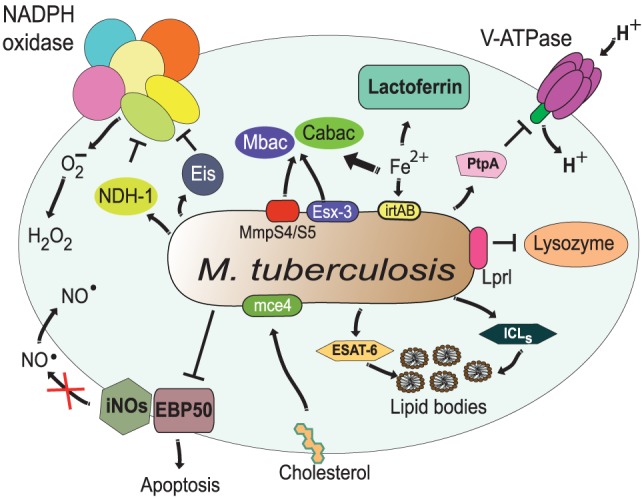
Resistance of *Mycobacterium tuberculosis* to phagolysosome contents. *M. tuberculosis* inhibits acidification by preventing the accumulation of V-ATPase on the phagosome membrane (161), in part through the action of protein tyrosine phosphatase (PtpA) (162). The bacterial lipoprotein, Lprl, can inhibit the lytic activity of lysozyme (226). The secretion system Esx-3 (230, 231) and the MmpS4/S5 transporters (232) are required for biosynthesis and secretion of the siderophores mycobactins (Mbac) and carboxymycobactins (Cabac), which seize Fe^2+^ from host proteins, such as lactoferrin (233). Then, the transporter system irtAB takes Fe^2+^ from Fe^2+^-carboxymycobactin into the bacterium (234, 235). The type I NADH dehydrogenase (NDH-1) (227) and the Eis protein (228) inhibit the NADPH oxidase, preventing formation of ROS. Also, *M. tuberculosis* prevents the generation of NO^⋅^ and apoptosis by interfering with EBP50, a scaffolding protein that controls the recruitment of iNOS at the membrane of phagosomes (229). In addition, *M. tuberculosis* alters the phagosome to divert host lipids for its own benefit through mce4, a cholesterol import system (236), and through accumulation of lipid bodies *via* the early secretory antigenic target-6 (ESAT-6) (237). The enzymes isocitrate lyases (ICLs) allow bacteria survival on even (acetate) and odd (propionate) chain fatty acids in lipid bodies (238).

Similarly, *M. tuberculosis* can resist in various ways the microbicidal components within the phagolysosome. A novel glycosylated and surface-localized lipoprotein, Lprl can inhibit the lytic activity of lysozyme (226) (Figure 8). Also, at least two proteins have been found to prevent the formation of ROS by inhibiting the NADPH oxidase. The type I NADH dehydrogenase (NDH-1) blocks ROS production to inhibit tumor necrosis factor alpha (TNF-α)-mediated host cell apoptosis (227) (Figure 8), while the enhanced intracellular survival (eis) gene product (Eis) abrogates production of both ROS and proinflammatory cytokines leading to arrest in apoptosis. These effects seem to depend on the *N*-acetyltransferase domain of the Eis protein (228) (Figure 8). In both cases, apoptosis is inhibited, but the mechanisms are different. In the case of NDH-1, apoptosis is dependent on caspase-3 and caspase-8 (227), while for Eis, apoptosis seems to be caspase independent (228). *M. tuberculosis* can also block RNS by interfering with EBP50, a scaffolding protein that controls the recruitment of iNOS at the membrane of phagosomes in macrophages. Interestingly, overexpression of EBP50 by a recombinant lentivirus had no effect on the iNOS recruitment to *M. tuberculosis*-containing phagosomes, but significantly increased the generation of NO^⋅^ and the level of apoptosis in macrophages (229). The EBP50-induced apoptosis was NO^⋅^-dependent and mediated by Bax and caspase-3 (229) (Figure 8). The mechanism for iNOS inhibition is not completely elucidated, but it seems to involve both having less iNOS on the membrane and blocking its enzymatic activity. The way *M. tuberculosis* prevents EBP50 functions remains a mystery.

Other pathogens are also known to display similar mechanisms against ROS and RNS. *Streptococcus agalactiae* (Group B *Streptococcus*) is an important cause of pneumonia and meningitis in neonatal humans (239). *S. agalactiae* expresses a superoxide dismutase (SodA), an orange carotenoid pigment, and glutathione. The latter two compounds functions as ROS scavengers (240, 241). *H. pylori* can also express a superoxide dismutase (SodB) (242), a catalase (KatA) (243), and the arginase RocF, which transforms the iNOS substrate arginine into urea (244, 245). Similarly, the yeast *C. albicans* expresses a copper and zinc containing superoxide dismutase (Sod1) (246), and a catalase (Cta1p) (247, 248), while *H. capsulatum* also secretes two catalases, CatB and CatP (249). The fungus *C. neoformans* produces a superoxide dismutase (250) and covers itself in a thick polysaccharide and melanin capsule that absorbs ROS (251). Also, the dimorphic fungus *Blastomyces dermatitidis* seems to be able to inhibit the enzyme iNOS to prevent the production of RNS (252). In all these pathogens, the expression of these enzymes and virulent factors effectively reduces the levels of ROS and RNS within the phagosome. Yet, very little is known about the mechanisms that induce expression of these virulent factors in each pathogen and the molecular details by which they inhibit NADPH oxidase and iNOS enzymes.

### Resistance to Nutrient Capture

The phagolysosome is a place where microbial nutrients are eliminated to arrest pathogen growth. As mentioned earlier, divalent cations, such as Fe^2+^, Zn^2+^, and Mn^2+^, are actively transported out of the phagolysosome (76). In response to this, several microorganisms have evolved mechanisms to retain these important nutrients. One strategy to acquire Fe^2+^ relies on the production of siderophores, which are low-molecular weight Fe^2+^-binding molecules of extremely high affinity, that remove Fe^2+^ from host proteins, such as hemoglobin, and transferrin (233). *S. aureus* produces two citrate-based siderophores, staphyloferrin A (SA) and staphyloferrin B (SB) (219, 220) (Figure 7). Together, SA and SB can trap enough Fe^2+^ to allow bacterial survival. These siderophores are very efficient because they avoid detection by the phagocyte siderophore-binding protein lipocalin (96, 97). In addition, *S. aureus* is also able to acquire Mn^2+^ through the action of Mn^2+^ transporters encoded by the bacterial gene loci *mntABC* and *mntH* (253). In *M. tuberculosis*, two groups of siderophores, mycobactins and carboxymycobactins, exist to overcome Fe^2+^ deficiency. The type VII secretion system Esx-3 contributes to siderophore production and release from these bacteria (230, 231) (Figure 8). Recently, another siderophore export system was identified in *M. tuberculosis*. The MmpS4 and MmpS5 transporters are required for biosynthesis and secretion of siderophores (Figure 8). Because a *M. tuberculosis* mutant lacking the mmpS4 and mmpS5 genes did not grow under low Fe^2+^ conditions and experienced Fe^2+^ starvation even under high-Fe^2+^ conditions, it seems that these transporters are the primary source of siderophores in mycobacteria (232). The importance of siderophore synthesis for Fe^2+^ acquisition is clear, but Fe^2+^ must find a way back into the bacteria. In *M. tuberculosis* an ABC transporter system, irtAB (product of the genes irtA and irtB), has been described for efficient utilization of Fe^2+^ from Fe^2+^ carboxymycobactin (Figure 8). Inactivation of the irtAB system decreases the ability of *M. tuberculosis* to survive Fe^2+^-deficient conditions (234, 235). Similarly, other microorganisms such as *A. fumigatus* (254) and *H. capsulatum* (255) can produce siderophores for Fe^2+^ capture.

Intracellular bacteria have also evolved various means to take nutrients from the host cell. Lipids are important building blocks for bacterial membrane formation and an energy source (256). Upon infection, *M. tuberculosis* alters the phagosome to divert host lipids for its own benefit. A virulent factor was identified within the gene cluster, mce4, because it was specifically required for bacterial survival during prolonged infection. It was found that mce4 encodes a cholesterol import system that enables these bacteria to derive both carbon and energy from this lipid in host membranes (236) (Figure 8). Also, mycobacteria-infected macrophages acquire a “foamy” phenotype characterized by the accumulation of lipid bodies, which serve as source of nutrients. This foamy phenotype is caused by bacterial manipulation of host cellular metabolism to divert the glycolytic pathway toward ketone body synthesis (237). This deregulation results in feedback activation of the anti-lipolytic G protein-coupled receptor GPR109A, causing changes in lipid homeostasis and accumulation of lipid bodies in the cell. ESAT-6, a secreted *M. tuberculosis* virulence factor, mediates the enforcement of this feedback loop *via* an unknown mechanism (237) (Figure 8). Another strategy used by *M. tuberculosis* to exploit host lipids involves the bacterial enzymes isocitrate lyases (ICLs). These ICLs are catalytically bifunctional isocitrate and methylisocitrate lyases that allow bacteria survival on even (acetate) and odd (propionate) chain fatty acids (238) (Figure 8). Moreover, the miR-33 induced by *M. tuberculosis* also inhibited fatty acid oxidation to support bacterial growth by a mechanism not yet described (177) (Figure 5). In addition, *M. tuberculosis* has yet another strategy to acquire lipids even from outside the cell in the lung environment. Alveolar macrophages are not only responsible for phagocytosis of these bacteria but also for catabolizing lung surfactant, a lipid–protein complex that lines the alveolar spaces. Recently, it was found that the scavenger receptor CD36 is redistributed to the macrophage cell membrane following exposure to surfactant lipids and participated in surfactant lipid uptake by these cells (180) (Figure 5). These macrophages also supported better bacterial growth in a CD36-dependent manner (180). Thus, it seems that CD36 mediates surfactant lipid uptake by human macrophages and that *M. tuberculosis* exploits this function for growth.

### Physical Escape from the Phagosome

In addition to resisting all the microbial effectors within a phagolysosome, several pathogens such as *C. neoformans, L. monocytogenes*, or *M. tuberculosis* can also completely escape from it. By getting out of the phagosome, these microorganisms can in the cytoplasm travel to other cell sites and finally leave the host cell.

**Figure 9:**
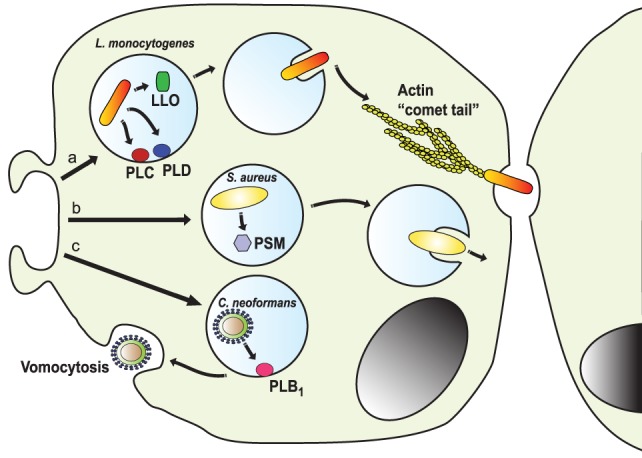
Escape from the phagosome. Several pathogens escape from the phagosome to persist in the less harsh environment of the cytoplasm. (a) The intracellular pathogen *Listeria monocytogenes* uses its virulent factor listeriolysin O (LLO) (264) and phospholipases (PLC and PLD) (265) to escape the phagosome. Once in the cytosol, the bacterium is propelled by the formation of actin “comet tails” that push it across the cell, allowing it to transfer between cells (266–268). (b) *Staphylococcus aureus* can escape from neutrophil phagosomes (269) by producing phenol soluble modulins (PSM), which are peptides with lytic activity toward many mammalian cells (270). (c) The fungus *Cryptococcus neoformans* escapes the cell by vomocytosis (257, 258). Here, the phagosome fuses with the cell membrane with assistance of the secreted phospholipase B1 (PLB_1_) (260), leaving the pathogen free and the phagocytic cell alive.

As mentioned earlier, the fungus *C. neoformans* is well equipped to replicate inside the phagosome. In addition, it can subsequently escape the cell by a non-lytic tactic known as vomocytosis (257, 258). Vomocytosis allows for the pathogen escape leaving the phagocytic cell alive (259). Although the molecular details of vomocytosis are not completely described, the process involves an exocytic fusion of the phagosome with the plasma membrane, thus releasing the fungus (259) (Figure 9). Vomocytosis also involves microtubules, but apparently not actin polymerization. Nevertheless, the formation of dynamic actin cages (“actin flashes”) around the phagosome is observed in many cases. These actin structures actually prevent vomocytosis. Yet, fungus strains with high rates of vomocytosis induce more actin flashes, suggesting that these flashes are a reaction from the cell to contain the phagosome. Still, at the end, the fungal phagosome is fused with the cell membrane and the pathogen is liberated (259). Also, the secreted phospholipase B1 (PLB_1_) is required for vomocytosis (260). It is thought that PLB_1_ helps permeabilizing the fungal phagosome to neutralize its lumen and to allow nutrients to come in (111, 261). Although vomocytosis is a unique escape function known only for cryptococci, a similar process has recently been described for *C. albicans* (262) and *Candida krusei* (263).

Another intracellular pathogen capable of escaping from the phagosome and then from the infected cell is *L. monocytogenes* (268). This bacterium uses its virulent factor listeriolysin O (LLO) to escape the phagosome (264) (Figure 9). LLO is a pore-forming toxin that permeabilizes the phagosome membrane. It is a potent toxin capable of also degrading the cell membrane, thus its expression and activity are strictly regulated. LLO expression is limited to the intraphagosomal phase of the bacteria, where it is induced by the low pH and high Ca^2+^ conditions of the phagosome (264). Also, LLO activation requires cooperation of host factor such as GILT (γ-inducible lysosomal thiol reductase) (271). In addition, several phospholipases are activated to completely degrade the phagosomal membrane and allow the bacterial escape (265). Once in the cytosol, the bacterium is propelled by the formation of actin tails that push it across the cell. This process is known as “actin rocketing” and it is initiated by the *Listeria* surface protein ActA (266, 267) (Figure 9). The actin fibers pushing the bacteria are called “comet tails” and propel the bacteria with enough force, allowing it to transfer between cells (268). In the same way, the Gram-negative bacteria *Shigella flexneri* can disrupt the phagosome membrane and escape into the cytosol (272), where it induces “comet tails” similar to *Listeria*. The bacterial protein IscA induces activation of N-WASp to initiate actin polymerization by the complex Arp2/3 (273). The actin “comet tails” then propel the bacteria across the cytosol and into neighboring cells. Bacteria from the genus *Rickettsia* are obligate intracellular pathogens that can also escape phagosomes. *Rickettsia* uses a secreted phospholipase A2 to disturb the phagosome membrane (274). Once in the cytosol, *Rickettsia* produce actin tails that allow them direct cell to cell transfer. The bacterial protein RickA is able to activate the Arp2/3 complex to initiate actin polymerization (275). Another microorganism that seems capable of phagosome escaping from neutrophils but not macrophages is *S. aureus* (269). These bacteria produce phenol soluble modulins (PSMs), which are peptides with lytic activity toward many mammalian cells (270). In particular, the α-PSM was found to induce a strong destruction of neutrophils after phagocytosis, allowing the escape of the phagocytized bacteria (276) (Figure 9).

Other bacteria, such as *M. tuberculosis* (277) and *Mycobacterium marinum* (278), can also escape phagosomes. After escaping the phagosome into the cytosol, *M. marinum* is able to move around by actin-mediated propulsion (279). The *M. marinum* actin tail formation involves activation of WASp proteins (280) and requires a functional region of difference 1 (RD1) loci (281). This RD1 locus encodes for a secretion system called the ESAT-6 system-1 (ESX-1) or type VII secretion system, which can induce pore formation on host-cell membranes (282). Thus, it was thought that all mycobacteria could escape from phagosomes using the pore-forming activity of ESX-1. However, this has to be formally proven experimentally. *M. tuberculosis* could be found in increasing numbers in the cytosol of dendritic cells and macrophages when infection was allowed to proceed beyond 2 days in culture (283), and the presence of cytosolic bacteria was also shown to occur *in vivo* (284). Therefore, there is no doubt about the capacity of mycobacteria to escape into the cytosol but the significance of this phenomenon is still a matter of debate. A simple idea is that bacteria need to leave the phagosome to replicate and then leave the cell. However, bacilli escape the phagosome at later times of infection and this is followed by cell lysis and release of bacilli (278). In consequence, escaping from the vacuole is not a requirement for either survival or growth of *M. tuberculosis* (285). Instead, it was proposed that the escape from the vacuole represents a transient state that could be critical to the rapid expansion of the bacterial population (285). If this is the case, then escaping from the phagosome is just an important step in the pathology that accompanies progression of tuberculosis infection to active disease. How, mycobacteria kill the cell to allow its release is not clear. Yet, recently, it was reported that the *M. tuberculosis* protein Rv3903c (channel protein with necrosis-inducing toxin, CpnT) is required for survival and cytotoxicity of *M. tuberculosis* in macrophages (286). CpnT consists of an N-terminal channel domain that is used for uptake of nutrients across the outer membrane and a secreted toxic C-terminal domain. This secreted portion is also named tuberculosis-necrotizing toxin (287). It can, in the cytosol of mycobacteria-infected macrophages, hydrolyze the essential coenzyme NAD(+) and induce cell necrosis. However, the mechanism for this cell lysis remains to be elucidated. Clearly, CpnT has a dual function in *M. tuberculosis*. It is used for uptake of nutrients within the phagosome and for induction of host cell lysis in the cytosol. The regulation of CpnT functions becomes then a topic of important research for controlling *M. tuberculosis* infections. Another *M. tuberculosis* virulence factor has also been found to participate in phagosome escape. The unique cell wall lipid phthiocerol dimycocerosates greatly augmented the bacteria escape from its intracellular vacuole (288), by a process not well understood. The mechanism for phagosome lysis is clearly complex as indicated by the fact that host molecules are also recruited by the bacteria to aid in its escape. Activation of host cytosolic phospholipase A2 rapidly led to phagosome lysis for bacteria moving into the cytoplasm of the host cell (116).

## Novel Therapeutic Opportunities

The study of the many mechanisms used by microbial pathogens to control phagocytosis provides opportunity for detecting novel potential targets of clinical intervention. Promising therapeutic approaches will be designed based on our new understanding of the tactics pathogens use to interfere with phagocytosis. For example, studies with miRNA in mycobacteria infections identified TLR2 as a potential target to prevent the blockage of phagosome maturation (179) (Figure 5). Recently, it was also found by gene expression profiling of human macrophages treated with glucocorticoids and/or IFN-γ that glucocorticoids, in contrast to IFN-γ, failed to trigger expression and phagolysosome recruitment of V-ATPase (289). This explained the increased risk for mycobacterial infections associated with the use of glucocorticoids. Moreover, this group also found that giving imatinib, a tyrosine kinase inhibitor, to glucocorticoid-treated macrophages induced lysosome acidification and antimicrobial activity without reversing the anti-inflammatory effects of glucocorticoids (289). Thus, an improved therapy would be to administer glucocorticoids together with drugs that induce phagosome acidification. In another recent report, a phagosome maturation assay using confocal microscopy in THP-1-derived macrophages infected with an attenuated *M. tuberculosis* strain was used to test the effects of Saxifragifolin D, a traditional Chinese medicine (290). Saxifragifolin D (a pentacyclic triterpenoid compound first isolated from the rockjasmine *Androsace umbellata*) reduced the inhibition of phagosome maturation. Using assays of this type, new potential drugs can be tested for future therapies.

Another potential therapeutic approach would be to modulate macrophage function to improve their antimicrobial potential against bacterial infections. The feasibility of such an approach has been suggested in a recent report of macrophage phagocytosis of *L. monocytogenes* (291). In this study, the engagement of receptor T cell immunoglobulin mucin-3 (Tim-3) on macrophages inhibited phagocytosis of *L. monocytogenes* by blocking nuclear erythroid 2-related factor 2 (Nrf2) signaling. In contrast, inhibition of Tim-3 augmented phagocytosis (291). Thus, modulating the Tim-3 pathway to alter macrophage function is a promising tool for treating infectious diseases, such as *Listeria* infections.

Phagocytosis of opsonized particles is, in general, more efficient and more efficacious in eliminating microorganisms. The idea to generate opsonizing antibodies for controlling infections is another promising area of opportunity for novel therapeutics. The value of this approach has been suggested in studies where opsonizing antibodies improve elimination of bacteria. In a study with five apparently healthy Indian donors having high titers of serum antibodies against *M. tuberculosis* cell membrane antigens, it was found that phagocytosis and killing of bacilli by the donor macrophages was significantly enhanced following their opsonization with antibody-rich, heat-inactivated autologous sera (292). Another study showed that antibodies directed at the R domain of *S. aureus* secreted coagulase could trigger phagocytosis and killing of staphylococci (293). This coagulase activates host prothrombin and generates fibrin fibers that cover the bacteria and prevent phagocytosis. These antibodies directed the fibrin-covered bacteria to phagocytes and also protected mice against lethal bloodstream infections caused by methicillin-resistant *S. aureus* isolates (293). Yet, another study, showed that a monoclonal antibody (mAb) directed at the Protein A could protect neonatal mice against *S. aureus* sepsis and create protective immunity against subsequent staphylococcal infection (294). A humanized version of this mAb was developed, and it is proposed as a potential new therapy for *S. aureus*-induced sepsis and meningitis in very-low-birth-weight infants (294). These reports encourage the development of novel vaccines that favor the formation of opsonizing antibodies against bacterial antigens to activate phagocyte innate immunity.

## Future Directions

Phagocytosis is a fundamental biological process (109) that in multicellular organisms is required for proper homeostasis and for fighting infections (1, 2). Therefore, it is not surprising that many microbial pathogens have mechanisms to counteract phagocytosis. As we have discussed here, for some model pathogens, namely *S. aureus* (295), *M. tuberculosis* (117), and *L. monocytogenes* (119), particular virulence factors that affect phagocytosis have been identified and to some extent the way they work is described. For many other microbial pathogens, their tactics for interfering with phagocytosis are only beginning to be defined. Despite the tremendous amount of published studies on microbial phagocytosis or knowledge on microbial control of this biological process is still incipient and fragmented. We know that some pathogens block phagocytosis at one step or another, but no information is available on how this blockage is accomplished. Some molecules have been identified but their mechanisms of action are not yet described. Future research will serve to fill these gaps and will provide clues on how to improve antimicrobial therapeutics.

An important element for future research is the implementation of novel techniques. Great advances have been achieved by application of proteomics analysis to phagosomes formed under different infection conditions (296). Earlier studies on *M. tuberculosis* phagosomes with high-resolution two-dimensional gel electrophoresis and mass spectrometry revealed unique bacterial proteins associated with the intracellular stage of the bacteria (297). The effect of a particular protein of the phagocytic machinery identified by proteomics can then also be tested by RNA-mediated interference (298). By comparing the protein profile of phagosomes formed with virulent and avirulent variants of a pathogen, relevant molecules for pathogenesis can be identified. For example, comparing phagosomes containing highly virulent *L. pneumophila* to phagosomes with avirulent *L. hackeliae* revealed a lack of Rho GDP-dissociation inhibitor (RhoGDI) in *L. pneumophila* replicative phagosomes (299). Similarly, comparing macrophage phagosomes formed after triggering different receptors, it was found that phagosome outcome was regulated by the individual receptors triggered for phagocytosis (300). This is in agreement with recent findings that indicate particular FcRs promote particular cell responses on neutrophil phagocytes (42). Thus, phagocytosis is clearly modified according to the receptor involved. We have a good understanding on how opsonic phagocytic receptors signal, but very little is known about the signaling pathways activated by other phagocytic receptors. This is an area of research that needs much further exploration in the future.

Other techniques that have been instrumental for our present understanding of phagocytosis are fluorescence microscopy coupled to particular probes to measure phagosome pH (301), to describe phospholipid dynamics during phagosome formation (302), and to quantify antibody-dependent phagocytosis (303). Together with these, the use of confocal microscopy coupled to fluorescence resonance energy transfer-based assays has been helpful to investigate the mechanisms of *L. monocytogenes* for phagosome escaping (304). Equally important, the use of novel microbial readouts of bacterial fitness have been developed to probe the host cell environments that promote or control bacterial growth (305). In particular, *M. tuberculosis* strains that express GFP under certain environmental signals relevant to the infection status of the macrophage, permitted identify infected phagocytes and demonstrated that bacteria in immune-activated phagocytes presented higher drug tolerance than bacteria in resting phagocytes (306). These assays will be very useful in future studies on phagocytosis of other microbial pathogens. To implement these assays, the proper fluorescent probes will need to be developed.

During phagocytosis, both the phagocyte and the microorganism adapt to fight and overcome each other. These changes, important to the final outcome of an infection, can be studied by modern techniques such as transcriptional analysis *via* RNA sequencing (RNA-seq). Changes in pathogen phenotype under various conditions are revealed when the total transcriptome is analyzed. For example, it is known that cigarette smoke predisposes exposed individuals to respiratory infections by enhancing the virulence of pathogenic bacteria. A recent study on the effect of cigarette smoke on *S. aureus* gene expression using RNA-seq revealed that these bacteria increased twofold the expression of protein A with the consequent reduction in phagocytosis (307). A similar comparative transcriptome study with RNA-seq of *Brucella melitensis* grown in normal-medium culture and in acid-medium (pH 4.4) culture revealed that 113 genes were differentially expressed. Among these genes, a two-component response regulator gene in the transcriptional regulation pathway was identified as important for acid resistance and virulence of *Brucella* (308). Also, an analysis of RNA-seq data from *in vivo* and *in vitro* cultures of *Cryptococcus gattii* identified highly expressed genes and pathways of amino acid metabolism that would enable these bacteria to survive and proliferate *in vivo* (309). Hence, particular genes expressed under particular conditions can be identified as potential therapeutic targets for controlling infections. Likewise, changes in cell phenotype can be analyzed by RNA-seq. For example, increased susceptibility to bacterial pneumonia is found after influenza infections. A recent RNA-seq analysis of alveolar macrophages revealed that the virus infection caused a reduction in the phagocytic receptor MARCO. This effect could be reversed after IFNγ treatment of monocyte-derived macrophages and THP-1 macrophages. Moreover, treatment with sulforaphane or SC79, activators of Nrf2 and Akt, respectively, caused increased MARCO expression and MARCO-dependent phagocytosis (310). Therefore, a promising strategy for controlling postinfluenza bacterial pneumonia would be to increase MARCO expression by targeting Nrf2 and Akt signaling in alveolar macrophages. Another example of RNA-seq analysis of macrophages in two different conditions, namely infection with virulent or avirulent strains of *M. tuberculosis*, revealed extensive remodeling of alternative splicing in macrophage transcriptome (311). This led to considerable increase in truncated/non-translatable variants of several genes with a decline in the corresponding protein levels. The product of one such gene, RAB8B that is required for phagosome maturation, was reduced due to elevated levels of truncated RAB8B variants in cells with virulent mycobacteria (311). Alternative splicing is a new mechanism that *M. tuberculosis* uses to control macrophages. The molecular details of this mechanism are not known and will certainly become an area of interesting research in the near future.

We have described phagocytosis as a general model based mainly on macrophages. However, there are important differences among diverse types of phagocytes and even between phenotypes of the same phagocyte. As indicated earlier, environmental cues can alter the functioning of a phagocyte, and no much is known about the mechanisms involved in these cell changes. Hence, this is an area of great interest, as shown by some recent studies. Metabolic conditions can alter macrophage function (312), and in the case of diabetes mellitus it was found that phagocytosis was reduced (313). This disease is also associated with increased tuberculosis risk and severity. Recently, it was also reported that alveolar murine macrophages from diabetic mice have a reduced expression of MARCO (314). The lack of this receptor could be the reason for inefficient phagocytosis in diabetic cells. Future research should determine whether other phagocytic receptors are also altered in diabetic macrophages. Nothing is known about the metabolic mechanisms that control phagocyte receptor expression.

The role of other phagocytes besides macrophages in controlling some intracellular bacterial infections is just beginning to be appreciated. For example, neutrophils also participate in controlling *M. tuberculosis* by autophagy (315) and are mobilized from the bone marrow to perform phagocytosis and secrete antimicrobial factors against *L. monocytogenes* (119). In addition, other cells such as dendritic cells can also perform phagocytosis by mechanisms that are different from those of macrophages (316). The particular role of these various phagocytic cells in different infection settings will also become an area of fruitful research in the future.

Macrophages not only perform phagocytosis of microbial pathogens but also ingest dead and dying host cells. The process of engulfing apoptotic cells is called efferocytosis, and it has an important role in the resolution of inflammation (317). Although efferocytosis of *M. tuberculosis*-infected cells leads to pathogen destruction, efferocytosis of *Leishmania*-infected neutrophils may promote infection (318). Understanding how macrophages, neutrophils, and dendritic cells process pathogens within a dying cell is another area for future research. Discoveries in this field should lead to novel therapeutics that simultaneously suppress inflammation and promote pathogen clearance.

## Conclusion

Elimination of pathogens by macrophages and neutrophils is an essential function of our innate defenses. These phagocytic leukocytes clear microorganisms from tissues *via* phagocytosis. Once inside the phagocyte, the microorganism is destroyed by a series of degrading mechanisms inside the phagosome. Despite this, many pathogens have evolved means to prevent phagocytosis or to resist its effects inside the phagocytic cells. Thus, these pathogens remain a considerable health threat. We have presented the main mechanisms phagocytes have for eliminating microbes and then we discussed the strategies used by some pathogens to interfere with each step of the phagocytic process. Our list of pathogens is not complete, since there are many microorganisms capable of resisting phagocytosis in ways, we do not completely recognize. Technical advances have allowed us to make significant advances toward understanding the molecular details of the interaction between some pathogens and phagocytes, but important questions remain. Future research in this area will certainly bring us interesting surprises that will help us conceive novel therapeutic approaches that could render pathogens more susceptible to phagocyte attack.

## Author Contributions

CR and EU-Q both equally conceived the issues, which formed the content of the manuscript, prepared the figures, and wrote the manuscript.

## Conflict of Interest Statement

The authors declare that this research was conducted in the absence of any commercial or financial relationships that could be construed as a potential conflict of interest.
